# Fasting and Its Impact on Skin Anatomy, Physiology, and Physiopathology: A Comprehensive Review of the Literature

**DOI:** 10.3390/nu11020249

**Published:** 2019-01-23

**Authors:** Nicola Luigi Bragazzi, Maha Sellami, Iman Salem, Rosalynn Conic, Mark Kimak, Paolo Daniele Maria Pigatto, Giovanni Damiani

**Affiliations:** 1 Postgraduate School of Public Health, Department of Health Sciences (DISSAL), Via Antonio Pastore 1, University of Genoa, 16132 Genoa, Italy; 2 Sport Science Program, College of Arts and Sciences (QU-CAS), University of Qatar, Doha 2713, Qatar; maha.sellami@gmail.com; 3 Department of Dermatology, Case Western Reserve University, Cleveland, OH 44106, USA; ims31@case.edu (I.S.); ruzica.conic@gmail.com (R.C.); mar.kimak@gmail.com (M.K.); 4 Clinical Dermatology, IRCCS Istituto Ortopedico Galeazzi, Milan, Italy; Department of Biomedical, Surgical and Dental Sciences, University of Milan, 20161 Milan, Italy; paolopigatto@valeo.it; 5 Young Dermatologists Italian Network (YDIN), Centro Studi GISED, 24121 Bergamo, Italy

**Keywords:** compliance and adherence to treatment, Ramadan or intermittent fasting, caloric restriction, skin disorders, chronotherapy and chronomedicine

## Abstract

Skin serves as the first protective line and barrier of the body. Like many other organs, skin can be affected by several disorders in response to external factors such as pathogens, ultraviolet light, and pollution, as well as endogenous alterations related to aging and/or oxidative stress disturbance. Researchers have reported new insights into how skin cells are altered in response to caloric restriction diets in mammals. One of the most well-known caloric restriction diets is the Ramadan intermittent fasting, which is a radical change in the diet plan of practitioners for the period of one lunar month. Ramadan fasting represents the fourth of the five pillars of the Islamic creed. Even though infirm individuals are waived to take part in this religious duty, patients with various health problems, including those with different skin disorders, might choose to share this event with peers and family members. No standardized protocols or guidelines exist, however, to advise their physicians on the proper management of their patients’ condition during fasting. With an increasing Muslim population living in Western countries, this topic has started to draw substantial attention, not only of Middle-Eastern physicians, but also of clinicians in the West. For this purpose, we carried out a comprehensive overview on the topic. Our main findings are that: (1) there is a strong need for evidence-based suggestions and guidance. Literature on the impact of the Ramadan fasting, as well as of other kinds of fasting, on skin diseases is scarce and of poor quality, as well as the information available from the Internet; (2) patients willing to fast should be advised about the importance of taking proper treatments or consider alternative options including administration of trans-dermal/topical drugs, as they are permitted during daylight hours. Further, non-compliance has important, clinical and economic implications for an effective patient management.

## 1. The Effects of Dieting on Health

**Figure 1:**
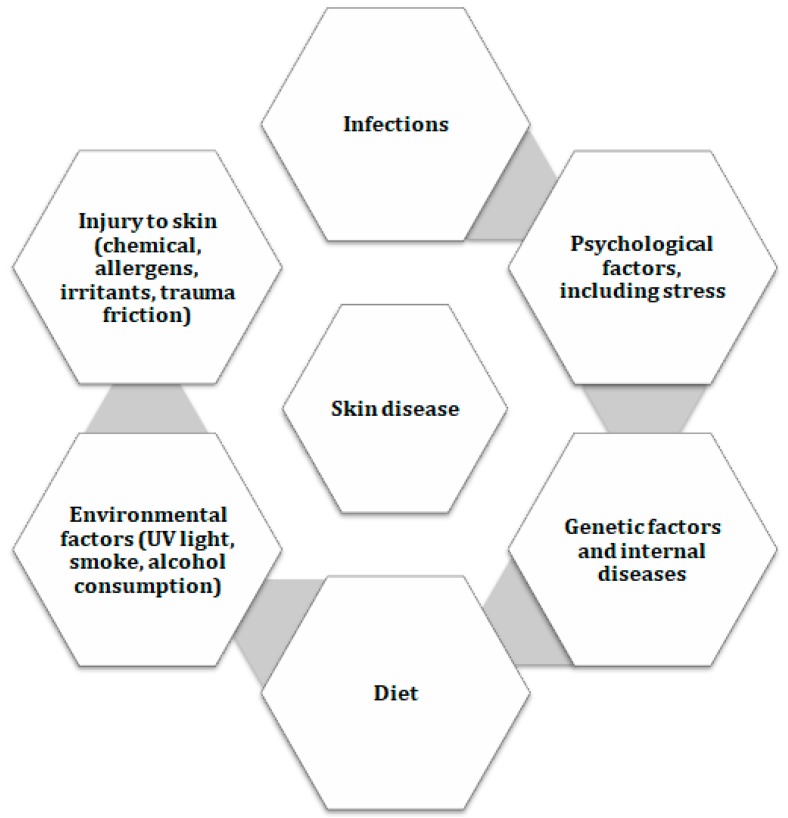
Factors affecting the skin. Abbreviations: UV (ultraviolet).

Skin disorders are commonly widespread skin conditions affecting both children and adults and may induce severe disability [1,2,3]. All skin conditions combined represent the fourth leading cause of non-fatal disability worldwide, especially in resource-poor regions [4]. In 2013, skin disorders were estimated to account for 1.79% of the total global burden of disease (GBD) calculated in disability-adjusted life years (DALYs) among 306 diseases and injuries [1,2]. These estimations are likely to differ among countries depending on the different healthcare systems and lifestyles adopted [5]. It is generally assumed that skin problems result from the interplay between various factors, including the biological make-up of the individual (genetic components, internal diseases, immunological susceptibility) and environmental factors (infections, stress, lifestyles, diet or exposure to ultraviolet (UV) light) [6] (Figure 1).

In recent decades, a huge body of research has investigated the impact of dieting on skin anatomy, physiology and physiopathology. As stated by Katta and Desai [7], the role of dietary manipulation as a potential non-pharmacological option for the treatment and management of skin diseases has been overlooked for a long time. Dieting can, instead, have various effects affecting health outcomes (as in the case of psoriasis) [8], modifying the prognosis (for example, in patients with acne) [9] or preventing or partially mitigating/counteracting the insurgence and the severity of the disorder (such as skin cancer and skin aging) [10].

Different dieting strategies/patterns and fasting protocols exist, based on (i) the degree of restriction of calorie intake (low-calorie, or very low-calorie dieting, when the daily food energy consumption is less than 800 kilocalories/day), (ii) the amount of hours of fasting (time-restricted feeding, intermittent or circadian fasting, alternate-day *versus* daily fasting), and (iii) the food or foods excluded (including low-fat, low-carbohydrate dieting, vegetarian or semi-vegetarian dieting) [11,12].

Emergent evidences seem to suggest that calorie restriction can protect against various diseases, including cancer, diabetes and heart disease. A calorie-restricted diet has been demonstrated to exert several beneficial effects, such as increasing lifespan, counteracting aging, modulating immune cell profile activity and reducing insulin resistance as well as preventing some stages of the carcinogenesis process [13]. 

Caloric restricted diets can also result in increasing the number of stem cells, which is a factor that plays a major role in tissue homeostasis and growth [14].

Dieting can also depend on personal dietary choices or on religious beliefs, such as the Adventist diet or the Ramadan fasting. While high-quality, large-scale epidemiological surveys, such as the “Adventist health study 1” (AHS-1) and the “Adventist health study 2” (AHS-2) [15], have investigated the impact of the Adventist diet on several health outcomes, the Ramadan fasting has been relatively understudied. 

The month of Ramadan is the ninth month of the Muslim lunar calendar (*al-Hijra*), which has great significance and value among Muslims, as it represents the month of the descent of the Noble Book (*al-Qu’ran*). The Ramadan fasting (*as-sawm*) is the fourth pillar of the Islamic faith (*arkan al-Islam*), together with the faith declaration or profession (*ash-shahada*) being the first pillar, the five daily ritual prayers (*as-salah*) as the second pillar, charity (*az-zakat*) (third pillar), and, finally, the pilgrimage to Mecca (*al-hajj*) for those who can afford it. The Ramadan fasting is not limited to abstinence from food and drinking, but also from smoking, systemic medication and sexual intercourse [16].

The Ramadan fasting is not, however, a continuous or prolonged fasting, but, rather, includes alternate fasting and feasting (re-feeding) periods [17], with a pre-dawn meal referred to as *suhoor*, and an after-sunset meal, called *iftar*. Since the Islamic calendar is a lunar one which is typically 11 days less than the Gregorian or solar calendar (354 and 365 days respectively), the duration of the Ramadan fasting can vary from 29 to 30 days, and the timing is 11 days earlier each year, therefore it can coincide with any season, which means that the fasting hours can sometimes exceed 18 hours, especially at higher latitudes [17,18].

Some Muslims such as pre-pubertal and pubertal children, menstruating women, pregnant and breast-feeding women, the elderly, sick people, and long distance travelers are waived from this religious duty [19]. However, they could choose to fast in order to share the spirituality of this month with their friends, peers and family members [20].

Some clinical implications of Ramadan fasting are well noted. Given the stress of fluid deprivation and subsequent electrolyte changes, kidney physiology is often significantly impacted. This is especially true among those with chronic kidney disease (CKD), predisposing them to acute tubular injury. Additionally, dehydration is a major precipitating factor for renal stone development [21,22,23].

Data on the effects of Ramadan fasting on skin disorders, however, is less well known and no standardized guidelines exist for advising optimal management methods and options. For this purpose, we have carried out this review to demonstrate possible clinical implications of the Ramadan fasting in terms of skin anatomy, physiology and physiopathology, warranting further investigation.

## 2. Anatomy and Physiology of the Skin 

Skin is the largest organ of the body, covering an impressive surface area of approximately 2 m^2^ and accounting for about 20% of total adult body weight. Being situated at the interface between the human body and environment, it is an important barrier, with both passive and active roles against chemical, physical and microbial insults [24].

Skin is composed of an upper layer (the epidermis), and a lower layer (the dermis) separated by a basement membrane. The epidermis is formed of 5 layers: the outermost layer is the *stratum corneum*, the *stratum lucidum* (present only in some specific parts of the human body, such as the fingertips, palms, and soles of the feet), the *stratum granulosum*, the *stratum spinosum*, and the *stratum basale* (the inner most layer that contains epidermal stem cells) [24].

The *stratum corneum* is particularly thick, consisting of dead cells (corneocytes) surrounded by lipid drafts representing the “formidable” physical barrier [24].

Dermis is divided according to the thickness of its collagen content into an upper *stratum papillare* and lower *stratum reticulare*, containing thin and thick collagen fibers, respectively [25].

Hypodermis or subcutaneous tissue is a layer of loose connective tissue and elastin that provides insulation from cold temperature, shock adsorbent capability, and a nutrient and energy storage reservoir [26]. The hypodermis is thickest in the buttocks, palms of the hands, and soles of the feet [27]. As we age, the hypodermis begins to atrophy, contributing to the thin wrinkled appearance of the aged skin [27].

Furthermore, skin harbors some innate and adaptive immune cells composing the skin immune system such as natural killers (NKs), mast cells, macrophages, highly specialized antigen presenting cells (APCs), epidermal dendritic cells (EDCs, also referred to as Langerhans cells), dermal dendritic cells (DCs, also known as interstitial or migratory DCs), αβ T cells, γδ T cells, and B cells [28,29], among others.

As such, the unique position and structure of the skin qualify it to perform different and peculiar functions including acting as a physical barrier, immune response, sensation (perception of pain, temperature, touch, and pressure), endocrine (vitamin D synthesis), neuro-endocrine (tightly networked to central stress axes), and homeostatic (expelling uric acid, ammonia, urea, and excess water) [29].

The skin has a natural self-healing ability [30]. When an aggression breaks or compromises the continuity of the cutaneous barrier, a healing process takes place in order to restore its integrity and preserve its function in about a week in the case of mild wounds. 

Scarring is a natural phenomenon but many factors can affect speed and quality, such as age and general condition of the individual, the cause of the injury, its depth or its location [30].

## 3. Epidemiology of Skin-Related Disorders in the Middle East and North Africa (MENA) Region

Skin diseases are considered among the most common clinical problems diagnosed in primary care settings in tropical areas and settings belonging to the Middle East and North Africa (MENA) region [31]. It should be noted that in these areas special fasting regimens, such as the Ramadan fasting, are adopted.

While several studies have investigated the prevalence and the economic burden of dermatoses among Arabs and Muslims living in the Middle East [32], little is known about the epidemiology of dermatological conditions among Arab Americans. 

In an interesting survey performed by Essawi et al. [33] in Michigan, the five most commonly self-reported skin conditions were eczema, acne, superficial fungal infections, melasma, and warts. The most concerning skin problem, however, was skin discoloration, uneven tone, and hirsutism. There were significant associations between socioeconomic status, time spent in the USA, and seeking medical advice.

## 4. Effects of Fasting on Skin Homeostasis

Different models of fasting have been studied in both animals and humans in an attempt to understand the effect of fasting on skin structure and function, including modified alternate-day fasting regimens [34], periodic diet mimicking fasting [35], short-term fasting [36], feed restriction [37], caloric or calorie or energy restriction or energy balance [38], or prolonged fasting [39], among others.

### 4.1. Fasting and Skin Structural and Functional Adaptation

It is plausible to assume that the skin, as the fundamental protective barrier against water and heat loss, microbial insults and mechanical injuries, plays a crucial role in the adaptation to limited caloric intake. In 2017, Forni et al. studied the skin’s adaptive structural and functional effects of mice exposed to long-term caloric restriction for 6 months. Authors reported statistically significant differences in the metabolic profile between the epidermis and dermis, with a more prominent oxidative metabolic profile in the dermis compared to the epidermis. This profile was associated with a marked increase in epidermal quiescent stem cells. In addition, there was an increase in inter-follicular stem cells which were believed to be responsible for maintaining fur and increasing the growth rates and retention of hair. Furthermore, there was an underdevelopment of the dermal adipocyte reservoir, an expansion of dermal vasculature, and an increase of vascular endothelial growth factor (VEGF). All the previous changes enabled the skin to maintain thermal homeostasis under conditions of restricted energy intake [40].

As previously mentioned, one of the main functions of the *stratum corneum* is to provide a permeability barrier to protect against excess water loss. Extracellular lipids formed primarily of ceramids, cholesterol, and fatty acids are the fundamental components of this barrier. The synthesis of cholesterol necessary for barrier formation takes place in the epidermis [41,42]. 

Wu-Pong and colleagues studied the effect of changes in plasma cholesterol levels on the synthesis of epidermal and dermal cholesterol and, subsequently, restoration of barrier function in hairless mice. Results have revealed a significant decrease in the cholesterologenesis in both layers with fasting resulting in a compromised barrier function which was not corrected by topical lipid application [41].

In a study evaluating the impact of caloric restriction on the side effects associated with topical retinoid treatment, there was a significant reduction in retinoid-induced skin irritation without interfering with the beneficial effects of the medication. The resultant mitigation of adverse events associated with fasting was attributed to two factors: the positive effect of caloric restriction on local antioxidant levels, and its inhibitory effect on the transcription of matrix metalloproteinase (MMP) genes involved in tissue destruction [42].

### 4.2. Fasting and Wound Healing

In an experimental mouse model, short-term fasting for 4 consecutive days repeated every 2 weeks for 2 months, followed by the induction of a cutaneous wound, was associated with an increase in wound healing compared to the control group. According to the authors, caloric restriction enhanced wound healing through the increase in macrophage activity. The production of transforming growth factor alpha (TGF-α) by macrophage during the re-epithelization phase of wound healing promotes keratinocyte proliferation. Additionally, macrophages also secrete VEGF, a potent angiogenic and fibrogenic factor necessary for granulation tissue formation [43].

Another study conducted by Hunt et al. in 2012, however, reported slower wound healing in a sample of 22 7-month-old Fischer-344 rats, 8 of which were maintained on a caloric restricted diet after wounding, compared to 5 controls which were fed *ad libitum*. This effect was corrected to a healing rate comparable with that of the control group when the caloric restricted rats were re-fed *ad libitum* for 2 days before wounding, in addition to maintaining a normal diet after wounding. This was explained by up-regulated expression of insulin-like growth factor-1 (IGF-1) binding protein 3 (IGFBP-3) and increased synthesis of type I collagen, resulting in enhancing the contractile capacity of the wound [44]. In other studies, a one-time fast for 72 hours with access to water only resulted in a decrease in cutaneous collagen formation.

In 2000, Miltyk and Palka postulated a fasting-associated decrease in pyrroline-5-carboxylate (P5C), a proline precursor molecule, to be responsible for the suppression of the IGF-1-dependent stimulation of collagen synthesis [45].

Another study conducted by Cechowska-Pasko et al. in 2003 attributed this effect instead to the decreased availability of IGF-1 for binding to its receptors, as a result of fasting-induced up-regulation in the levels of the phosphoisoform of IGF-I binding protein type 1 (IGFBP-1), known for its high binding affinity to IGF-1 [46]. In 2004, another hypothesis was adopted by the same research group. They proposed the decline in prolidase activity, an enzyme responsible for proline salvage from imidodipeptides for re-use in collagen synthesis, as an explanation for the negative effects of fasting on collagen biosynthesis. They reported that inhibition of prolidase activity could be attributed to a fasting-associated reduction in pyruvate kinase (PK) enzyme activity resulting in the accumulation of a strong prolidase inhibitor factor (PIF), namely the phosphoenolpyruvate (PEP) [47].

**Figure 2:**
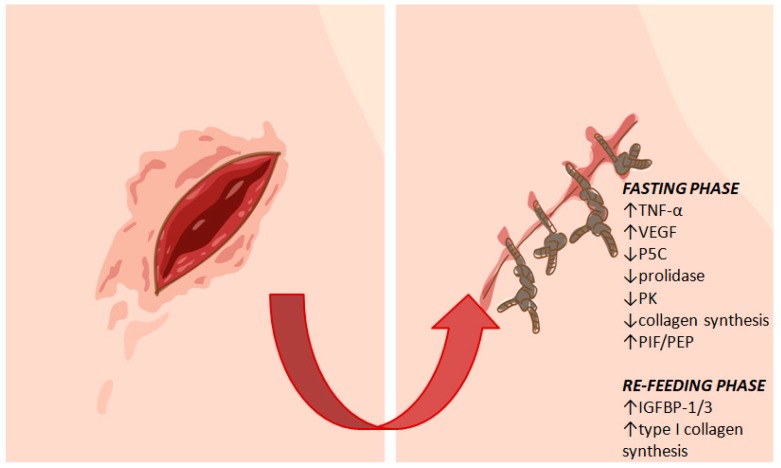
The hypothesized mechanisms of the biphasic effect of intermittent fasting on wound healing. Abbreviations: IGFBP-1 (insulin-like growth factor binding protein 1); IGFBP-3 (insulin-like growth factor binding protein 3); P5C (pyrroline-5-carboxylate); PEP (phosphoenolpyruvate); PIF (prolidase inhibitor factor); PK (pyruvate kinase); TNF-α (tumor necrosis factor alpha); VEGF (vascular endothelial growth factor).

Figure 2 pictorially shows the hypothesized mechanism of fasting effect on wound healing.

### 4.3. Fasting and the Immune System 

Some studies investigated the effects of prolonged fasting for a minimum of 3 days followed by re-feeding and have shown a favorable outcome on the immune system. The decreased level of circulating IGF-1 and protein kinase A (PKA) signaling induced by prolonged fasting resulted in modulation of long-term hematopoietic stem cells (HSCs) promoting self-renewal, lineage regeneration and proliferation, especially of NKs, and stress resistance, an effect believed to be protective against the toxic effect of chemotherapy on HSCs in humans. Additionally, it was proven that short-term starvation can enhance the phagocytic activity of macrophages promoting the process of wound healing and providing protection against some granulomatous infections [48].

### 4.4. Fasting and Skin Growth Regulation

IGF-1 as a mitogen is known for its pro-growth effects which include suppression of apoptosis, increased angiogenesis, and stimulation of cell proliferation. IGFs are produced in most cells, with IGFBPs that modulate their actions, and act locally on a paracrine or autocrine mode. IGF stimulates proliferation (especially IGF-1) and cell differentiation. Because of their homology structure with insulin, IGFs can, under determined pharmacological doses, act on the insulin receptor. In particular, IGF-1 can lower blood sugar, inhibit the processes of lipolysis and of catabolism of proteins. The mitogenesis downstream signaling cascade of IGF-1 involves the activation of mitogen-activated protein kinase (MAPK) and phosphatidylinositol 3-kinase (PI3K) pathways [49,50,51]. In 2007, Xie et al. studied the effect of 20% dietary caloric restriction for 5 days a week extended through 10 weeks with and without exercise on the development of skin tumors in “SENsitivity to CARcinogenesis” (SENCAR) mice, known for their sensitivity to 12-*O*-tetradecanoylphorbol-13-acetate (TPA)-induced skin cancer. The research group recorded a decrease of Ras-MAPK and PI3K-Akt pathways, in addition to down-regulation of the expression of 31 and 34 genes related to MAPK and PI3K pathways, respectively. This was explained by a significant reduction of IGF-1 levels associated with dietary restriction, suggesting a potential anti-carcinogenic role [52].

### 4.5. Fasting and Skin Aging

Skin aging is a complex, multi-factorial process characterized by decreased collagen level (in particular, collagen type 1, which lowers the ratio of collagen type 3/collagen type 1, and collagen type 7), loss of fibrillin-positive biostructures and broken elastin.

As previously stated, caloric restriction without deficiency of essential nutrients has been linked to enhancement of lifespan and decreased aging [53]. The non-enzymatic glycation and oxidation processes can affect various body proteins including, but not limited to, plasma proteins, hemoglobin, and skin collagen. The accumulation of glycoxidation products such as carboxymethyl lysine (CML) and pentosidine in cutaneous collagen promotes skin aging. A classic study conducted by Cefalu et al. in 1995 evaluated the effect of a long-term 60% caloric restriction program on the levels of CML and pentosidine in rodent skin. Authors found that chronic caloric restriction decreased the glycation rate of skin proteins, resulting in the reduction of age-related accumulation of these metabolites in cutaneous collagen [54]. On the other hand, the effect of telomere attrition on restricting cell replication capacity and subsequently its substantial role in aging is well established, even though its role in fasting remains controversial. Although telomere length (TL) is inversely related to body mass index (BMI), telomere dynamics did not seem to be affected by caloric restriction [55].

**Figure 3:**
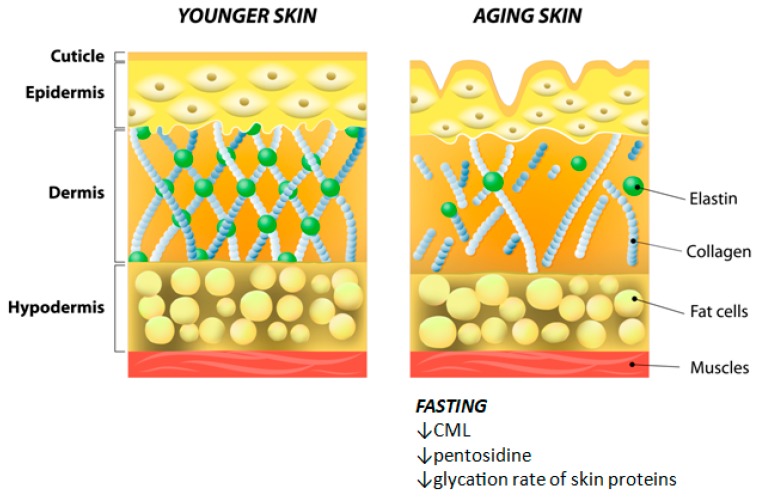
The anti-aging effect of fasting. Abbreviations: CML (carboxymethyl lysine).

Figure 3 pictorially shows the hypothesized mechanism of fasting on skin aging.

### 4.6. Fasting and Skin Effects: A Summary

**Figure 4:**
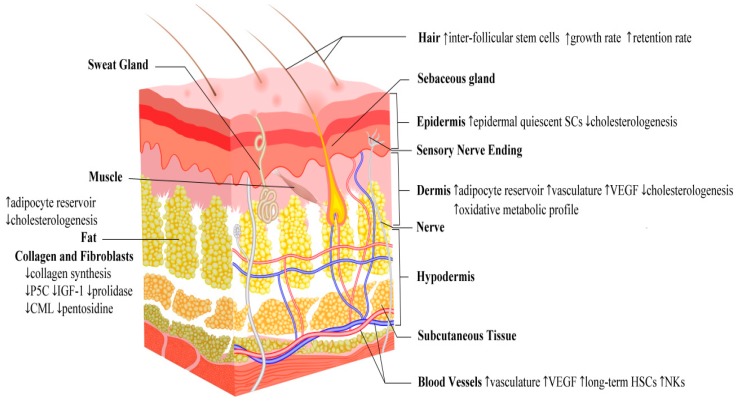
The major effects of fasting on skin anatomy, homeostasis dynamics and physiology. Abbreviations: CML (carboxymethyl lysine); HSCs (hematopoietic stem cells); IGF-1 (insulin-like growth factor 1); NKs (natural killers); P5C (pyrroline-5-carboxylate); SCs (stem cells); VEGF (vascular endothelial growth factor).

Taking together all the previously mentioned mechanisms, it can be concluded that caloric restriction/fasting has an important impact on skin. Figure 4 reports the main effects on skin anatomy, homeostasis dynamics, and physiology, whereas Table 1 synthesizes animal models and experiments of different fasting regimens showing the major effects of caloric restriction on skin.

## 5. Fasting and Autoimmune/Inflammatory Dermatoses

In addition to its significant role in the course of many metabolic and gastrointestinal disorders such as diabetes and inflammatory bowel disease (IBD) [56,57], dietary restriction can also influence the progression of skin diseases [58], such as atopic eczema, psoriasis, and acne [59].

Lithell et al. reported an improvement of two chronic inflammatory dermatoses, atopic dermatitis and *pustulosis palmaris et plantaris*, with intermittent fasting for two weeks. The results were associated with a low concentration of unsaturated iron and lactoferrin, known for their anti-apoptotic effects on neutrophils [60,61].

Other studies also indicated the amelioration of psoriatic lesions following caloric restriction. This was imputed to the modulator effects of fasting on the immune system such as a decrease in the activity of pro-inflammatory clusters of differentiation 4 (CD4) positive T helper (Th) cells and an increase in anti-inflammatory cytokines secretions like IL-4, resulting in dampening of inflammation [62].

Another study performed by Smith et al. in 2008 has shown the beneficial impact of caloric restriction on *acne vulgaris* lesions. This was explained by decreased sebum production, which thereby counterbalances one of the main factors in the pathogenesis of *acne vulgaris* [63]. Sebaceous glands are small oil-producing glands present in the skin of mammals and produce sebum. Excess sebum results in surface oiliness and blocked pores, providing nourishment to bacteria that live on the skin (in particular, *Propionibacterium acnes* or *P. acnes*) and contributes to acne flare-ups. Some emergent studies seem to support the idea that diet and acne may be, at least in part, connected. Some studies found a strong relationship between a fasting type diet and acne in human adults and young subjects. In fact, during the period of caloric restriction, sebum level was found to be reduced by 40%, which influenced the degree of acne severity [64,65]. However, these results were observed during severe caloric restriction (<100 calories per day) with a reversed increase following normal diet.

*Prurigo pigmentosa* (PP), first described by Nagashima et al. in 1971, is defined as a rare inflammatory skin disorder, characterized by itchy, reticulated, and erythematous plaques or papules. A possible role of the ketoacidotic status associated with fasting and dietary restriction has been implicated in the pathogenesis of the disease.

Hijazi and collaborators identified 4 cases of PP with the help of the dermatopathology database; 3 out of these patients reported the coincidence of their skin condition with fasting [66].

## 6. Fasting and Skin Cancer

The impact of fasting regimen on skin cancer has been investigated by some studies. For instance, Corazzari et al. explored the effect of the combination of antiblastics (such as cisplatin) with calorie restriction protocols (nutrient deprivation) in models of wild type and mutated BRAFV600E melanoma cell lines. Fasting was found to increase the sensitivity of tumor cell lines to cisplatin-induced cells, and also of those cell lines particularly resistant to any pharmacological treatment. From a mechanistic standpoint, cell death (but not autophagy) accounted for this effect: more in detail, apoptosis was fostered by the reactive oxygen species (ROS) accumulation and expression of the Activating transcription factor 4 also known as ATF4 in the absence of endoplasmic reticulum-stress. Moreover, authors found that exposure to 2-deoxy-*D*-glucose further increased this effect in a model of SK Mel 28 cell lines [67].

In another study, fasting was found to modulate the IGF-1 receptor (IGF-1R)/epithelial growth factor (EGF) receptor (EGFR) and the Akt/mTOR pathways, which are dysregulated in obesity and may lead to skin cancer [68].

## 7. Ramadan, Chronotherapy and Skin

Every cell of the body is involved in a state of adaptation in order to maximize its function and cope with challenges associated with the circadian rhythm. Not surprisingly, skin has cycles of physiological and functional changes highly influenced by the internal master clock [69,70,71]. The circadian rhythm is mastered by the suprachiasmatic nucleus (SCN) of the hypothalamus stimulated by light entering the retina; however, due to the unique position of the skin and its exposure to the external environment, it is plausible to consider the skin as a peripheral clock receiving various stimuli such as humidity, UV, pollutants, and changes in temperature. Other peripheral clocks include muscles, fat and liver, among others.

Accordingly, many physiological properties of the skin, including trans-epidermal water loss (TEWL), hydration, capillary blood flow, temperature, sebum production, and keratinocyte proliferation, undergo periodicity during the day [69,70,71]. This, indeed, supports the use of a “chronotherapeutic approach” in the systemic administration as well as the topical application of certain medications to maximize the therapeutic effects and minimizing the adverse reactions. For example, the barrier function of the skin is more compromised at night with an increase in TEWL, cutaneous blood flow, adaptive immune activity, release of pro-inflammatory cytokines and histamine, while the secretion of corticosteroid typically decreases, which can explain the reported circadian rhythm of some dermatoses, in particular inflammatory and itchy diseases. Therefore, the typical timing for treatments like emollients, anti-inflammatory drugs including corticosteroids, and anti-histamines is typically in the evening [71,72,73,74,75,76,77,78,79,80,81].

Similarly, since the proliferation of epidermal stem cells is higher during sleep hours, the ideal timing for immuno-modulators such as retinoids, azathioprine, methotrexate, and Apremilast is the evening to optimize their anti-inflammatory and anti-proliferative effects and minimize their side effects as seen in decreasing the gastrointestinal and hematological adverse reactions in a mouse model with a night dose of mycophenlate mofetil. Biological therapy is best administrated in the evening to counterbalance the night surge of pro-inflammatory cytokines [82,83].

Changes in the sleep-wake cycle was reported with intermittent fasting during the holy month of Ramadan, particularly in the first two weeks. A study by BaHammam and colleagues in 2010 has investigated the alterations in circadian rhythms among six healthy Muslim men with flexible working hours through the month, using sensor devices. Interestingly, all the subjects reported a shift of their sleep cycle, so that they were mainly sleeping during the day and working and eating during the night. This was, indeed, accompanied by a delay in the acrophase of their cutaneous temperature, as well as their energy expenditure [84].

Accordingly, this change in the circadian rhythm should be taken into consideration when prescribing medications to the patients; however, a problem of potential non-compliance could be encountered since, for cultural habits and beliefs, the oral administration of these medications during the day will be considered as a break in the fasting. Furthermore, the problem of non-adherence is not only limited to systemic drugs, but some Muslims even avoid the use of topical medications during the Ramadan fasting. A prospective survey, investigating the development of dermatological disorders during the Ramadan fasting, was carried out by Patel and co-authors at one of the tertiary hospitals in the UK. The study included 35 men and 40 women. The authors reported the eruption of cutaneous disorders such as eczema in 13 individuals, psoriasis, acne vulgaris and psoriasis in 8 patients, in addition to vitiligo, rosacea, urticaria and hair loss. Interestingly, more than one third of the participants denied the use of any topical treatment during fasting, which they considered breaching of their fast. This quite widespread belief was not limited to specific age group, gender, socioeconomic status, or educational level [85].

## 8. Future Prospects

Dietary interventions/manipulations represent a promising approach for treating, managing and, at least partially, preventing skin disorders. Despite such important practical implications, this topic has been neglected in the existing scholarly literature, when it deserves further research. High-quality randomized controlled trials (RCTs) should be conducted to systematically explore and compare different fasting protocols, including the use of vegetables and fruits for caloric and metabolic manipulations. For instance, consumption of foods rich in polyphenols protects against UV and exerts photoprotective effects, counteracting or mitigating UV-induced skin inflammatory status, proliferation, DNA damage and dysregulation of several cellular networks and pathways, including immune responses [86].

## 9. Conclusions

Despite the fact that the month of Ramadan represents a living laboratory in which different working hypotheses (anti-aging, anti-carcinogenesic, and pro-wound healing effects of fasting) can be tested *in vivo*, its importance for clinical investigations that could have broad, significant translational implications has been overlooked.

In particular, the following knowledge gaps can be listed:

(1) There is a strong need for evidence-based suggestions and guidelines. Literature on the impact of the Ramadan fasting as well as of other fasting regimens on skin diseases is scarce and of poor quality, as well as information available from the Internet. Instead, chronotherapy and chronomedicine should be taken into account and further explored;

(2) Few studies have been conducted, recruiting small convenience samples, with high non-responder rates;

(3) The impact of the Ramadan fasting on skin health could be compared with the effect of other kinds of fasting, including periodic diet, calorie restriction, dietary restriction, dietary manipulation, intermittent, short-term, and prolonged fasting.

However, despite the dearth of studies on the topic, based also on our clinical experience, the following recommendations can be made:

(1) No serious risks for health have been so far reported and, therefore, patients willing to fast should be advised about the importance of continuing their treatment and that administration of trans-dermal/topical drugs is licit during the Ramadan fasting;

(2) Non-compliance and non-adherence can have important clinical and economic implications for patient management; therefore, patient education and empowerment play a major role;

(3) Physicians should be instructed in recognizing rare dermatological disorders associated with fasting, such as PP;

(4) Further larger, high-quality studies are still needed in order to fill in the above-mentioned knowledge gaps.

## Figures and Tables

**Table 1:** Summary of animal models and experiments showing different effects of various fasting models on skin homeostasis dynamics.

Animal Model	Fasting Regimen	Results	Explanation	References
Swiss Mice	Long term caloric restriction to 60% for 6 months	Maintained/preserved thermal homeostasis	Maintenance of fur due to increase of HFSC pool Expansion of dermal vasculature due to increased levels of VEGF	Forni et al., 2017 [38]
Hairless Mice	Caloric restriction	Compromised *stratum corneum* permeability barrier	Decreased synthesis of epidermal cholesterol	Wu-Pong et al., 1994 [41]
UM-HET3 Mice	Caloric restriction to 70% for 3–18 months	Decreased retinoid-induced skin irritation without interfering with treatment efficacy	Increased local antioxidant levels Inhibitory effect on transcription of MMP genes involved in tissue destruction	Varani et al., 2008 [42]
Suri Mice	Short-term fast for 4 day/2weeks for 2 months	Enhancement of wound healing	Increased MQ production and release of TNF-α and VEGF	Hayati et al., 2011 [43]
Fisher-344 Rats	40% calorie restricted diet begun 48 hours before wounding and continued during healing	Delayed wound healing	Decreased collagen synthesis	Hunt et al., 2012 [44]
Rats	One time 72-hour fast with access to water only	Delayed wound healing	Decreased level of the proline precursor, P5C, resulting in suppression of IGF-1 dependent stimulation of collagen synthesis	Miltyk and Palka, 2000 [45]
Rats	One time 72-hour fast with access to water only	Delayed wound healing	Decreased availability of IGF-1 due to up-regulation of IGFBP-1 that has high affinity to IGF-1	Cechowska-Pasko et al., 2003 [46]
Rats	One time 72-hour fast with access to water only	Delayed wound healing	Decreased prolidase activity leading to decreased proline salvage and reduction of collagen synthesis	Cechowska-Pasko et al., 2004 [47]
Mice	2-day water-only fast before chemotherapy	Protective effect against toxic effects of chemotherapy on HSCs	Decreased levels of IGF-1 and PKA, resulting in modulation of HSC, promoting self-renewal, lineage regeneration and proliferation	Cheng et al., 2014 [48]
SENCAR Mice	20% calorie restricted diet for 5 days/week for 10 weeks	Anti-carcinogenic role	Decrease in mitogenesis downstream signaling cascade of IGF-1 (PI3K-AKT and Ras-MAPK)	Xie et al., 2007 [52]
Rodent	Long term 60% caloric restriction	Decreased aging	Decreased concentration of glycoxidation products (CML and pentosidine) in cutaneous collagen	Cefalu et al., 1995 [54]

Abbreviations: CML (carboxymethyl lysine); HFSC (hair follicle stem cells); HSC (hematopoietic stem cells); IGF-1: insulin-like growth factor 1; IGFBP-1 (insulin-like growth factor binding protein 1); MAPK (mitogen activated protein kinase); MMP (matrix metalloproteinase); MQ (macrophages); P5C (pyrroline-5-carboxylate); PI3K-AKT (phosphoinositide3-kinase-protein kinase B); PKA (protein kinase A); TNF-α (tumor necrosis factor alpha); VEGF (vascular endothelial growth factor).
